# Endometrial “Scratching” An update and overview of current research

**DOI:** 10.4274/jtgga.galenos.2020.2019.0175

**Published:** 2020-06-08

**Authors:** Veronika Günther, Sören von Otte, Nicolai Maass, Ibrahim Alkatout

**Affiliations:** 1 Clinic for Obstetrics and Gynecology, UKSH Campus Kiel, Kiel, Germany; 2 University Fertility Center, Ambulanzzentrum des UKSH gGmbH, Kiel, Germany

**Keywords:** Infertility, reproductive immunology, perinatal immunology and inflammation

## Abstract

About one in every six couples is affected by sterility. Assisted reproduction procedures are currently the treatment of choice for a number of patients who desire children. Many causes of sterility can be overcome with the aid of in vitro fertilization, but successful implantation of the embryos is the major limiting factor. Failure of implantation may occur repetitively. In the treatment of sterility, many approaches have been used to overcome the barrier of implantation failure and improve the chances of successful nidation. Scratching the endometrium prior to embryo transfer has been suggested as one means of enhancing the likelihood of implantation. The current literature was examined to investigate if there was any possible benefit from endometrial scratching. The studies were divided according to whether the women suffered from recurrent implantation failure or not. In summary, it was found that unselected subfertile women generally benefit less from endometrial scratching, but scratching appears to be successful in women who have experienced repeated implantation failure. Although the heterogeneous body of data on the subject deserves further clarification. The latest data presented at “European Society of Human Reproduction and Embryology” 2018 in Barcelona suggested that the method should be abandoned.

## Introduction

Approximately one in every six couples is affected by sterility. Procedures of assisted reproduction are currently the method of choice for a number of patients desiring children (1). Many causes of sterility can be overcome by the aid of in vitro fertilization (IVF), but successful implantation of the embryo is still the major limiting factor. Implantation, also known as nidation, starts on day 5 and ends on day 10, post conception (p.c.). The zona pellucida surrounding the blastocyst ruptures (on day 4 p.c.) due to growth of the blastocyst and enzymatic lysis; this phenomenon is also known as hatching. This is followed by apposition and adhesion of the blastocyst to the endometrium. In this process the microvilli on the surface of the external trophoblast cells interact with the epithelial cells of the uterus and form junctional complexes with the aid of surface glycoproteins (2). An essential element of implantation is the estrogen- and progesterone-induced reconstruction of the endometrium during the luteal phase, which is responsible for the receptivity of the endometrium. This episode, which is limited to a period of a few days, is referred to as the implantation window (3).

## Recurrent implantation failure

Implantation failure may occur repeatedly. Recurrent implantation failure has been variously defined. One assumes a failure of pregnancy after two to six IVF cycles, following the transfer of at least 10 embryos of good quality (4). Other authors refer to repeated implantation failure when no clinical pregnancy has occurred after the transfer of at least four embryos in at least three fresh embryo transfers (ET) or cryo-thawed ETs; it is assumed that the patient is below 40 years of age (5). There are many possible causes that may be responsible for the infertility of the couple. Uterine factors such as polyps or uterine abnormalities, infections, thrombophilia, immunological factors or genetic factors are just a few examples. In this context, endometriosis, which is particularly frequently associated with an increased sterility rate, deserves special mention. The prevalence of endometriosis in female infertility patients, at 25-50%, is significantly higher than in fertile women. It is assumed that 30-50% of endometriosis patients are confronted with sterility (6). Endometriosis remediation is associated with an increased pregnancy rate and should be performed prior to planned fertility treatment (7,8).

## Endometrial “scratching”

**Figure 1:**
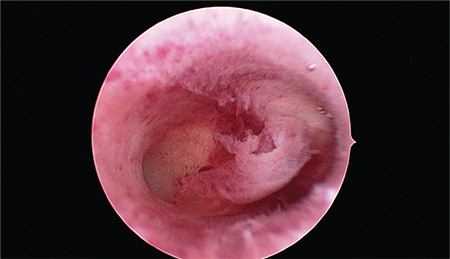
Hysteroscopy Hysteroscopic view of an inconspicuous cavum uteri. In the middle of the picture the thrown-up endometrium is shown and in the rear part the exit of the right tube can be seen

Several approaches have been used to overcome the problem of implantation failure in the treatment of sterility, or improve the chances of successful nidation. Scratching of the endometrium prior to ET is one method of enhancing the likelihood of implantation. Usually, in the luteal phase of the cycle preceding IVF, the endometrium is “scratched” with a small catheter, 3 mm in width, known as the Pipelle^®^. Usually without hooking on the cervix, the catheter is pushed forward through the cervix to the fundus, and then retracted in circular movements in order to stimulate the endometrium (Figure 1). In case further diagnostic investigation is desired, such as the investigation of chronic endometritis or the presence of plasma cells or uterine killer cells, the Pipelle^®^ can be used simultaneously to obtain a biopsy specimen of the endometrium. This is regarded as a low-risk procedure with a low rate of complications. Scratching can be performed on an outpatient basis without anesthesia and is associated with minor pain for the majority of patients.

**Figure 2:**
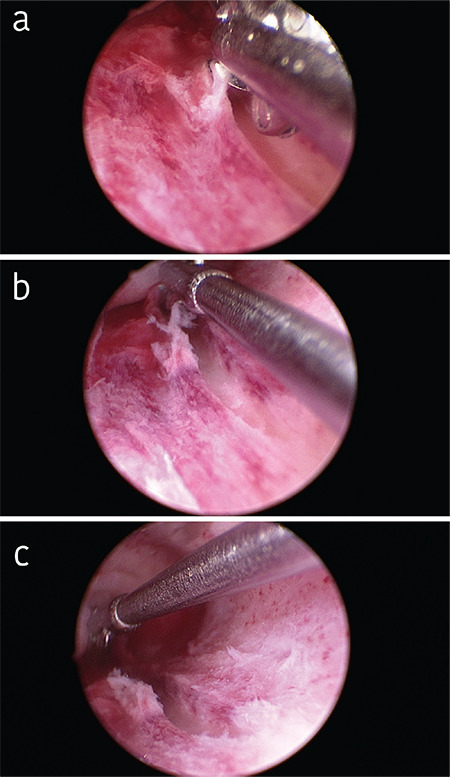
a, b, c) Endometrial scratching For immunomodulatory stimulation, a mucosal lesion is created usually on the posterior wall with the hysteroscope

As an alternative to the use of the Pipelle^®^, scratching can also be performed in the course of a diagnostic hysteroscopy. In order to evaluate the uterine cavity and detect or rule out potential barriers to implantation, such as a septum, a polyp, or a myoma, it is usually sufficient to perform a mini-hysteroscopy. Figure 1 shows the hysteroscopic view of an inconspicuous cavum. In this intervention the gynecologist may perform an endoscopy of the uterine cavity without anesthesia, and usually even without hooking the cervix. The small optical instrument, measuring just 3 mm in diameter, serves the purpose of inspection as well as “scratching” or endometrial stimulation. After inspection and photographic documentation, when the instrument is withdrawn, a mucosal lesion is created usually on the posterior wall (Figure 2a, b,c).

The first observations about scratching were made in 1907 by Loeb (9), who described the rapid proliferation of decidual cells after injury to the endometrium in the uterus of guinea pigs. In 2003 Barash et al. (10) first reported injury to the endometrium and its positive effect on implantation rates. They showed that a biopsy of the endometrium on day 8, 12, 21, and 26 of the menstrual cycle was associated with a higher pregnancy rate after IVF. Endometrial injury resulted in the secretion of growth factors and cytokines during the healing process, which, according to the authors, exerted a positive effect on endometrial receptivity (10).

Three approaches have been used to enhance uterine receptivity by endometrial scratching and thus enhance pregnancy rates after IVF-ET:

1. Local stimulation of the endometrium which induced decidualization, which in turn increases the likelihood of implantation of the transferred embryo (11).

2. The healing and repair process after successful scratching caused a significant increase in macrophages, dendritic cells, and proinflammatory cytokines, including tumor necrosis factor-alpha (TNF-α), growth-regulated oncogene-α, and macrophage-inflammatory protein-1B (MIP-1B), which exert a positive effect on implantation (11,12). Especially TNF-α and MIP-1B were found in high concentrations during the implantation window, which underlined the inflammatory effect on the receptive endometrium (13).

3. Ovarian stimulation during IVF treatment has been associated with high levels of estrogen, which causes an early increase in progesterone levels. Compared to the embryonic stage, the endometrium is already in an advanced stage of differentiation, which makes it difficult for implantation to take place (14,15,16). Scratching during the preceding cycle may suppress proliferation and thus optimize synchronicity between the endometrium and the embryo to be transferred (11).

## Current data

A number of studies and overview articles have focused on scratching and success rates of subsequent pregnancies.

## Studies on recurrent implantation failure

A meta-analysis and review in 2012 comprised 2062 women from four randomized and three non-randomized controlled studies; one to six IVF attempts had been made prior to the study. A hysteroscopy in the early proliferative phase as well as endometrial scratching in the preceding cycle, interpreted as endometrial injury, were regarded as inclusion criteria. The evaluation revealed a 70% higher rate of clinical pregnancies in the intervention group compared to the control group (17). Karimzadeh et al. (18), and Narvekar et al. (2), who were also included in the above mentioned meta-analysis, noted higher pregnancy rates after successful screening in their respective randomized controlled studies.

Karimzadeh et al. (18) included 58 patients in the treatment group and 57 patients in the control group. Implantation rates were 10.9% in women who underwent scratching and 3.4% in controls (p=0.039); pregnancy rates were 27.1% and 8.9%, respectively (p=0.023). No difference was noted in miscarriage rates (p>0.05). Narvekar et al. (2) included 49 patients in the treatment group and 51 women in the control group, both after recurrent implantation failure. Scratching was performed once in the follicular phase and a second time in the luteal phase, both in the cycle preceding IVF. It should be noted that, in the control group, a hysteroscopy was performed on day 7-10 of the preceding cycle; the hysteroscopy might have caused mild mechanical stimulation and also effected an alteration of the endometrium (19). Implantation, clinical pregnancy, and live birth rates were significantly higher in the intervention group than in controls (implantation rates 13.07% vs 7.1%; clinical pregnancy rates 32.7% vs 13.7%, p=0.01; live birth rates 22.4% vs 9.8%; p=0.04) (2).

Shohayeb and El-Khayat (20) showed that scratching performed during hysteroscopy resulted in significantly higher implantation, pregnancy, and live birth rates compared to hysteroscopy without scratching. Two hundred patients with recurrent implantation failure were included in the study, and were assigned to the treatment and control groups in equal numbers. Group A received a hysteroscopy in the early follicular phase (day 4-7), with endometrial scratching of the fundus and the posterior wall, whereas group B only underwent a diagnostic hysteroscopy (21). Implantation rates were 12% in group A, and just 7% in group B (p=0.015). Clinical pregnancy rates were 32% in group A and 18% in group B (p=0.034). Live birth rates were 28% in group A and 14% in group B (p=0.024). Miscarriage rates did not differ significantly (12.5% in group A and 22% in group B; p=0.618) (20).

In a randomized controlled study, Kumbak et al. (22) investigated the outcome of IVF after hysteroscopy and endometrial biopsy on day 21 of the cycle during the luteal phase. A sample was obtained with a small biopsy catheter and sent for histological investigation. Seventy patients in the treatment group were compared with 58 patients in the control group; the latter had received no intervention. Pregnancy rates were significantly higher in the treatment group (82% vs 73%; p=0.009) than in controls. Given the same number of transferred embryos of category A, the implantation rates (38% vs 25%, p=0.04) and pregnancy rates per embryo (67% vs 45%; p=0.01) were significantly higher in the scratching group than in controls (22).

In two further randomized controlled studies, the authors registered no benefit from scratching (23,24). Baum et al. (23) performed a randomized double-blind study comprising 36 patients who had undergone at least three previous IVF attempts. The intervention group (n=18) underwent scratching twice (day 9-12 and day 21-24), followed by IVF treatment. The special feature of the control group (n=18) was that the patients underwent a placebo investigation during which the biopsy catheter was inserted into the cervix without contacting the endometrium. The study revealed lower implantation rates (2.08% vs 11.1%, p=0.1), clinical pregnancy rates (0% vs 31.25%, p<0.05), and live birth rates (0% vs 25%, p=0.1) in the treatment group compared to controls.

A more recent randomized controlled study performed in 2015 by Gibreel et al. (24) also revealed no statistically significant improvement in live birth rates after scratching compared to controls. A subgroup analysis, on the other hand, showed a higher live birth rate in women who had undergone two or more failed IVF attempts after scratching, compared to those who had undergone only one IVF attempt (24).

A Cochrane analysis performed by Nastri et al. (25) in 2015 comprised 14 studies with a total of 1063 patients in the treatment group and 1065 patients in the control group. Endometrial scratching was performed between day 7 of the preceding cycle and day 7 of the ET cycle. The control group underwent no manipulation of the endometrium. A prerequisite was at least two previous ETs. Higher rates of pregnancies and live births were noted in the intervention group (relative risk: 1.42; 95% confidence interval: 1.08-1.85, p=0.01). A subgroup analysis, which excluded all studies with a potential bias, yielded an equally significant result. If 30% of women who underwent no scratching had become pregnant, the intervention group would have achieved a pregnancy rate of 33-48% (25). Scratching, according to the authors, had no impact on miscarriage rates, potential bleeding, or multiple pregnancies (25).

In contrast to the above mentioned studies, the following authors investigated the impact of scratching in women without recurrent implantation failure.

## Studies on women without recurrent implantation failure

In a prospective randomized study comprising 121 women who had undergone IVF treatment, Zhou et al. (26) performed endometrial scratching in the intervention group (n=60) when they noted irregular endometrial patterns in the vaginal ultrasound investigation (atypical, absence of trilaminar pattern, echogenic lesions). Scratching was performed during ovarian stimulation in all cases, with the purpose of enhancing endometrial receptivity. The control group underwent no scratching. The treatment group revealed higher implantation rates (33.33% vs 17.78%), clinical pregnancy rates (48.33 vs 27.86%) and live birth rates per ET (41.67% vs 22.96%) after scratching (26).

Nastri et al. (27) performed scratching with a Pipelle^®^ 7 to 14 days prior to scheduled hormonal stimulation for an IVF cycle, while the women were taking an oral contraceptive. The authors registered higher pregnancy and live birth rates (p=0.01) in the treatment group compared to the control group, with no impact on miscarriage rates (p=0.53) (27).

Guven et al. (28) achieved similar results, although they performed scratching on day 3 of the transfer cycle rather than the preceding cycle. The authors registered higher pregnancy (48.2% vs 29%, p=0.025) and live birth rates (33.9% vs 17.7%, p=0.035) in the treatment group compared to controls (28).

In contrast to the majority of authors, who investigated the effect of scratching by local manipulation in the preceding cycle, Karimzade et al. (29) investigated the effect of scratching with the Novak curette on the day of follicle aspiration. One hundred fifty-six patients were included in this prospective controlled study. However, this study revealed negative effects on implantation rates (7.9% vs 22.9%, p=0.002) and the outcome of IVF (9.6% vs 29.1%, p=0.004) after scratching compared to controls. It may be assumed that, since manipulation was performed shortly before ET, pro-inflammatory cytokines, macrophages and dendritic cells could not be formed rapidly enough in adequate numbers. The receptivity of the endometrium was damaged rather than enhanced as a result thereof (29).

In their treatment group (n=50) Safdarian et al. (30) performed scratching with a biopsy catheter on day 21 of the preceding cycle, and registered no statistically significant difference compared to controls (n=50) in regard of implantation and pregnancy rates (30).

Yeung et al. (31) achieved similar results. In their randomized controlled study the authors included 300 subfertile women, selected randomly, who were scheduled to undergo or had undergone IVF cycles. In the treatment group the authors performed scratching with a Pipelle^®^ in the mid-luteal phase of the preceding cycle. Compared to controls, the authors registered no differences in regard of implantation, pregnancy, multiple pregnancy, or miscarriage rates. In a subgroup analysis of women who had undergone repeated IVF attempts, the pregnancy rate after scratching was lower than that in controls (31).

In a recent but retrospective case control study performed in May 2017 in Israel (32), 238 patients were included in the treatment group and 238 in the control group. Women in the treatment group underwent scratching for the first time. Scratching was performed once or twice in the proliferation phase as well as the luteal phase. The results in the scratching and control groups were similar in regard of implantation (28.06% vs 30.08%, p=0.8), pregnancy (34.03% vs 40.33%, p=0.18), and continued pregnancy rates (18.48% vs 28.99%, p=0.33) (32).

At the European Society of Human Reproduction and Embryology meeting, which was held from 1 to 4 July 2018 in Barcelona, Dr. Sarah Lensen from New Zealand presented the recent results of her work on the subject of scratching (33). Her contribution received the Clinical Science Award for oral presentation. Meanwhile her work has been published in the New England Journal of Medicine (34). Data from this randomized multicenter study were collected between June 2014 and June 2017 at 13 centers in five countries. 1364 women (690 in the scratching arm vs 674 in the control group) who had undergone ET after IVF during the fresh embryo or cryo-thawed cycle were included in the study, with no recent exposure to disruptive intrauterine instrumentation (e.g., hysteroscopy). Participants were randomly assigned in a 1:1 ratio to either endometrial scratching (by pipelle biopsy between day 3 of the cycle preceding the embryo-transfer cycle and day 3 of the embryo-transfer cycle) or no intervention. The primary outcome was live birth. The results revealed no increase in live birth rates after endometrial scratching: 26.1% (180/ 690) vs 26.1% (176/674) in controls, odds ratio 1.00 (0.78-1.27). Even a subgroup analysis in regard of recurrent implantation failure, fresh or cryo-thawed cycles, and the interval between scratching and ET yielded no specific group that would benefit from scratching. The authors concluded that endometrial scratching should not be offered or performed in the course of fertility treatment.

## Conclusion

### Practical significance

To estimate the final value or benefit of scratching in regard of implantation, pregnancy, and live birth rates, it is important to precisely define the respective patient population that would benefit from this intervention. Scratching is able to enhance the receptivity of the endometrium, but a number of other pathologies may be responsible for implantation failure. The present overview of studies shows that unselected, sub-fertile women generally benefit less from endometrial scratching. In contrast, scratching appears to be a successful measure for enhancing the chances of implantation in women with recurrent implantation failure. However, recent data from Lensen et al. (34) contradict this thesis. Rather, these data have shown that endometrial scratching is not associated with a higher live birth rate even in women with recurrent implantation failure. Patients should be informed of these recent data.

Scratching is convenient, easy to perform, and associated with very little pain. Based on the existing body of data, as mentioned above, scratching could be offered to patients with recurrent implantation failure in order to try to enhance pregnancy and live birth rates, after the women have been informed in detail about the procedure. The patients should definitely be informed of the heterogeneous data on the subject including the most recent work from Lensen et al. (34) Taking these latest data into account, endometrial scratching did not show any advantage in pregnancy and live birth rate and the conclusion of this work was that this method should not now be offered, which patients should be made aware of.
